# Meeting abstracts from the Annual Conference on Hereditary Cancers 2015: Szczecin, Poland. 24-25 September, 2015

**DOI:** 10.1186/s13053-017-0074-9

**Published:** 2017-10-10

**Authors:** Ella R. Thompson, Michelle Wong-Brown, Simone M. Rowley, Susan Dooley, Na Lil, Michael Hipwell, Simone McInerny, Cliff Meldrum, Lisa Devereux, David Mossman, Alison H. Trainer, Briar-Rose Millar, Gillian Mitchell, Cate Smith, Paul A. James, Ian G. Campbell, Rodney J. Scott, Katarzyna Klonowska, Anna Jakubowska, Jelena Maksimenko, Arvids Irmejs, Miki Nakazawa-Miklasevica, Inga Melbarde-Gorkusa, Genadijs Trofimovics, Janis Gardovskis, Edvins Miklasevics, Karolina Tęcza, Jolanta Pamuła-Piłat, Joanna Łanuszewska, Ewa Grzybowska, Edvins Miklasevics, M. Szwiec, J. Tomiczek-Szwiec, M. Gełej, C. Cybulski, T. Huzarski, E. Kilar, Małgorzata Oczko-Wojciechowska, Michał Świerniak, Jolanta Krajewska, Małgorzata Kowalska, Tomasz Tyszkiewicz, Agnieszka Pawlaczek, Michał Jarząb, Monika Kowal, Dagmara Rusinek, Jadwiga Zebracka-Gala, Agnieszka Czarniecka, Barbara Jarzab, Andrzej Plawski, Paweł Borun, Joanna Szczepinska, Monika Siolek, Beata Kozak-Klonowska, Katarzyna Kaczmarek, Magdalena Muszyńska, Wojciech Marciniak, Grzegorz Sukiennicki, Marcin Lener, Katarzyna Durda, Katarzyna Jaworska-Bieniek, Tomasz Gromowski, Tomasz Huzarski, Tomasz Byrski, Jacek Gronwald, Oleg Oszurek, Cezary Cybulski, Tadeusz Dębniak, Antoni Morawski, Anna Jakubowska, Jan Lubiński, Grzegorz Sukiennicki, Magdalena Muszyńska, Wojciech Marciniak, Katarzyna Kaczmarek, Marcin Lener, Katarzyna Durda, Katarzyna Jaworska-Bieniek, Tomasz Gromowski, Tomasz Huzarski, Tomasz Byrski, Jacek Gronwald, Oleg Oszurek, Cezary Cybulski, Tadeusz Dębniak, Antoni Morawski, Anna Jakubowska, Jan Lubiński, Michał Post

**Affiliations:** 1 Cancer Genetics Laboratory, Peter MacCallum Cancer Centre, East Melbourne, Victoria Australia; 2 Department of Oncology, University of Melbourne, Melbourne, Victoria Australia; 3 The University of Newcastle and Hunter Medical Research Institute, Newcastle, Australia; 4 Division of Genetics, Hunter Area Pathology Service, Newcastle, Australia; 5 Hereditary Cancer Program, BC Cancer Agency, Vancouver, Canada; 6 European Centre for Bioinformatics and Genomics, Institute of Bioorganic Chemistry, Polish Academy of Sciences, Poznan, Poland; 7 Department of Genetics and Pathology, Pomeranian Medical University, Szczecin, Poland; 8 Oncology Institute, Riga Stradins University, Riga, Latvia; 9 Center for Translational Research and Molecular Biology of Cancer, Maria Sklodowska-Curie Memorial Cancer Center and Institute of Oncology, Gliwice Branch, Gliwice, Poland; 10 Institute of Oncology, Riga Stradins University, Riga, Latvia; 11 Regional Oncology Center, Opole, Poland; 12 Department of Genetics and Pathology, International Hereditary Cancer Center, Pomeranian Medical University, Szczecin, Poland; 13 Department of Oncology, Disctict Specialist Hospital, Świdnica, Poland; 14 Department of Nuclear Medicine and Endocrine Oncology, Maria Skłodowska-Curie Memorial Cancer Center and Institute of Oncology, Gliwice Branch, Gliwice, Poland; 15 III Department of Radiotherapy and Chemotherapy, Maria Skłodowska-Curie Memorial Cancer Center and Institute of Oncology, Gliwice Branch, Gliwice, Poland; 16 Genomic Medicine, Medical University of Warsaw, Warsaw, Poland; 17 Institute of Human Genetics, Polish Academy of Sciences, Poznan, Poland; 18 Department of Genetics, Holycross Cancer Centre, Kielce, Poland; 19 Department of Genetics and Pathology, International Hereditary Cancer Center, Pomeranian Medical University, Szczecin, Poland; 20 Read – Gene, S.A., Grzepnica, Poland; 21 Department of Genetics and Pathology, International Hereditary Cancer Center, Pomeranian Medical University, Szczecin, Poland; 22 Read – Gene, S.A., Grzepnica, Poland; 23 Department of Ophthalmology, Pomeranian Medical University, Szczecin, Poland

## A1 Panel Testing for Breast Cancer Risk Assessment: is it just because we can rather than should?

### Ella R. Thompson^1,2^, Michelle Wong-Brown^3^, Simone M. Rowley^1,2^, Susan Dooley^4^, Na Li1^2^, Michael Hipwell^4^, Simone McInerny^1,2^, Cliff Meldrum^4^, Lisa Devereux^1,2^, David Mossman^4^, Alison H. Trainer^1,2^, Briar-Rose Millar^4^, Gillian Mitchell^2,5^, Cate Smith^1,2^, Paul A. James^1,2^, Ian G. Campbell^1,2^, Rodney J. Scott^3,4^

#### ^1^Cancer Genetics Laboratory, Peter MacCallum Cancer Centre, East Melbourne, Victoria, Australia; ^2^Department of Oncology, University of Melbourne, Melbourne, Victoria, Australia; ^3^The University of Newcastle and Hunter Medical Research Institute, Newcastle, Australia; ^4^Division of Genetics, Hunter Area Pathology Service, Newcastle, Australia; ^5^Hereditary Cancer Program, BC Cancer Agency, Vancouver, Canada

Since the identification of BRCA1 and BRCA2 a number of other genes have been reported to be associated with an increased risk of breast cancer. Many of these genes have now appeared on commercial massively parallel sequencing (MPS) panels and are increasingly used for assessing breast cancer risk in women who developed disease at unusually young ages. Apart from BRCA1 and BRCA2 where there is considerable evidence associated with disease risk as well as strategies to mitigate the effects of mutation carriage there is little if any information about the consequence mutations in the more recently identified breast cancer susceptibility genes. This includes knowledge about the histopathology conferred by the loss of expression of a particular gene, the influence of environmental factors on disease risk as well as the most effective treatment strategies for disease prevention or disease treatment. For many of the genes listed on commercial panels knowledge about disease frequency in affected populations compared to control populations is lacking thereby undermining the veracity of breast cancer susceptibility claims.

To help address the shortfall in information about some of the more recently identified genetic risk factors to breast cancer we undertook a study of 2000 cases and 2000 controls to estimate the prevalence of mutations in a panel of genes that are commonly included in commercial testing. The results reveal that MPS panel testing must continue under a research setting so that more information can be gathered to understand what is meant by the term “genetic predisposition” to breast cancer for a large proportion of genes that are currently under scrutiny. At present only four genes can be used unequivocally in a diagnostic setting for the assessment of genetic risk of disease in most countries.

## A2 Analysis of the association of *APOBEC3B* deletion with familial breast and/or ovarian cancer risk in Polish population

### Katarzyna Klonowska

#### European Centre for Bioinformatics and Genomics, Institute of Bioorganic Chemistry, Polish Academy of Sciences, Poznan, Poland

AID/APOBEC family consists of 11 cytidine deaminases that possess a capability of introducing sequence alterations in DNA and RNA. Proteins from AID/APOBEC family are involved in various cellular processes, including antibody diversification and innate response against retroviruses. Some of the AID/APOBEC members (primarily APOBEC3A and APOBEC3B) were also reported to be possible mutagenic enzymes responsible for induction of specific hypermutation patterns in several cancer types, including breast cancer. Recently, an association of common CNV in the *APOBEC3* cluster (deletion of the *APOBEC3B* gene) with breast cancer risk was reported in both Chinese and European populations (OR~1,3). Our project is aimed at the characterization of the *APOBEC3B* deletion structure, development of molecular tests for the deletion genotyping and analysis of the deletion contribution to familial risk of breast and/or ovarian cancer. Firstly, we performed a sequencing analysis of the *APOBEC3B* deletion breakpoints. Based on the identified breakpoints, we designed a simple PCR-based test that allows for genotyping of the deletion in a large group of DNA samples. Additionally, we designed MLPA assay that was utilized for the independent confirmation of structure and genotypes of *APOBEC3B* deletion. We subsequently used the designed tests for the analysis of the *APOBEC3B* deletion in groups of patients with familial breast and/or ovarian cancer, patients with unselected ovarian cancer, and controls. Preliminary results confirmed association of *APOBEC3B* deletion with breast cancer and indicated that the deletion may contribute to aggregation of breast and/or ovarian cancer cases in family. Due to low frequency of *APOBEC3B* deletion in Polish population (~6%) the obtained results are only marginally significant and have to be replicated in larger groups of cases and controls. Both PCR-based test and the designed MLPA assay will be freely available to research community and can be used to study the *APOBEC3B* deletion in other populations or other cancer types.

**Acknowledgments**

NCN 2011/01/B/NZ5/02773, MNiSW 500-8045.

## A3 Constitutional methylation of BRCA1 gene as breast cancer risk factor

### Anna Jakubowska

#### Department of Genetics and Pathology, Pomeranian Medical University, Szczecin, Poland

It has been proposed that methylation signatures in blood-derived DNA may correlate with cancer risk. In this study, we evaluated whether methylation of the promoter region of the BRCA1 gene detectable in DNA from peripheral blood cells is a risk factor for breast cancer, in particular for tumors with pathologic features characteristic for cancers with BRCA1 gene mutations. We conducted a case-control study of 66 breast cancer cases and 36 unaffected controls. Cases were triple-negative or of medullary histology, or both; 30 carried a constitutional BRCA1 mutation and 36 did not carry a mutation. Blood for DNA methylation analysis was taken within three months of diagnosis. Methylation of the promoter of the BRCA1 gene was measured in cases and controls using methylation-sensitive high-resolution melting (MS-HRM). A sample with any detectable level of methylation was considered to be positive. Methylation of the BRCA1 promoter was detected in 15 of 66 cases and in 2 of 36 controls (OR 5.0, *p* = 0.03). Methylation was present in 15 of 36 women with breast cancer and without germline BRCA1 mutation, but in none of 30 women with breast cancer and a germline mutation (*p* < 0.01). The association between methylation and breast cancer was restricted to women with no constitutional BRCA1 mutation (OR 12.1, *p* = 0.0006). Methylation of the promoter of the BRCA1 gene detectable in peripheral blood DNA may be a marker of increased susceptibility to triple-negative or medullary breast cancer.

## A4 Prognostic role of BRCA1 mutation in patients with triple-negative breast cancer

### Jelena Maksimenko, Arvids Irmejs, Miki Nakazawa-Miklasevica, Inga Melbarde-Gorkusa, Genadijs Trofimovics, Janis Gardovskis, Edvins Miklasevics

#### Oncology Institute, Riga Stradins University, Riga, Latvia

Triple-negative breast cancer (TNBC) is proposed to be an immunohistochemical surrogate of the basal-like breast cancer subtype. In spite of the relative chemosensitivity of this cancer subtype, it is characterized by aggressive clinical behavior; therefore, a further subclassification of TNBC is required to develop new targeted treatment. In previous studies, a strong correlation between BRCA1 mutation-associated tumors and TNBC has been identified. The aim of the present study was to investigate the prognostic significance of carrying two germline BRCA1 founder mutations (4153delA and 5382insC) in patients with TNBC in the Latvian population. A total of 78 consecutive BRCA1 mutation-negative and 38 BRCA1 mutation-positive invasive TNBC patients in stage I–IV with no history of ovarian or other primary advanced cancers, who had undergone definitive surgery and genetic testing between 2005 and 2011, were deemed eligible for study. Relapse rates and breast cancer-specific survival (BCS) outcomes were compared between mutation carriers and non-carriers. Univariate and multivariate analyses Cox proportional-hazards models were used to compute independent predictors of survival outcomes. No statistically significant differences were identified in relation to tumor size, T stage, stage, Ki-67 status and tumor differentiation grade between the two groups. The median follow-up period was 36 months for mutation carriers and 41 months for non-carriers. A higher proportion of BRCA1 mutation non-carriers experienced distant recurrence compared with that of mutation carriers (P<0.03). BRCA1 mutation carriers had a significantly higher BCS than non-carriers (94.9 vs. 76.9%; P<0.02). In the univariate analyses, BRCA1-positive status was associated with decreased risk of distant recurrence (HR, 0.228; 95% Cl, 0.052–0.997; P<0.049) and breast cancer-specific mortality (HR, 0.209; 95% Cl, 0.048–0.902; P<0.036). In the multivariate analysis Cox proportional-hazards model, BRCA1-positive status was an independent favorable prognostic factor for distant recurrence-free survival (HR, 3.301; 95% Cl, 1.102–9.893; *P*<0.033). In conclusion, results of the present study demonstrate that positive BRCA1 founder mutation status in TNBC, with no evidence of ovarian or other cancer type in advanced stage, significantly improves prognosis.

## A5 Genetic determinants of response to FAC chemotherapy in breast cancer patients

### Karolina Tęcza^1^, Jolanta Pamuła-Piłat^1^, Joanna Łanuszewska^1^, Ewa Grzybowska^1^

#### ^1^Center for Translational Research and Molecular Biology of Cancer, Maria Sklodowska-Curie Memorial Cancer Center and Institute of Oncology, Gliwice Branch, Gliwice, Poland

Clinical resistance to breast cancer chemotherapy is observed as incidences of disease progression, local recurrence, primary and secondary tumors at different locations, and cancer-related mortality. Apart from tumor-related factors, chemotherapy resistance is associated with patient’s body abilities to metabolize and remove drugs from the system. The factors that can influence the drug’s therapeutic potential are reduced transport of drugs into tumor cells, overexpression of efflux transporters and modified DNA repair systems which remove drug-induced damage.

Primary endpoint of our study was to identify the genetic and clinical determinants of non-responsiveness to FAC chemotherapy in breast cancer patients. The analyses were conducted in the group of 324 women from the Silesian voivodeship diagnosed with breast cancer. Genes and their polymorphic variants were selected on the basis of analyses of their known or potential role in transport and metabolism pathways of all three FAC drugs, as well as in DNA damage repair systems. The genotyping was performed for 22 genetic variants in 17 genes- ABCB1, ABCC2, ABCG2, ATM, MTHFR, DPYD, GSTT1, GSTM1, GSTP1, CYP1B1, CYP2C19, TYMS, ERCC1, ERCC2, XRCC1, TP53, SLC22A16.

The results revealed that the presence of preexisted metastases and the variants c.-24C>T (rs717620) in ABCC2 gene, p.Lys751Gln (rs13181) in ERCC2 gene and p.Ser893Ala/Thr (rs2032582) in ABCB1 gene were the independent prognostic factors of treatment responsiveness. Cumulative analysis shown that the growing number of high-risk genotypes is reflected in gradual increase in risk of non-responsiveness to treatment- from OR 2,68 (95% CI 1,37-5,23; *p*=0,004) for presence of two genotypes to OR 9,93 (95% CI 1,28-77,25; *p*=0,027) for carriers of all three negative genotypes. The growing number of unfavorable genotypes was also reflected in shortening of treatment failure-free survival from 54,4 months or carriers of one variant, to 51,5 and 34,9 months for the carriers of two and three genotypes, respectively. Furthermore, the division of patients based on the number of unfavorable genotypes revealed the subgroup of triple negative breast cancers, carriers of all three high-risk genotypes, that harbor an extremely high risk of FAC treatment non-responsiveness (OR 34,0; 95% CI 1,20-967,50; *p*=0,028).

Our results emphasize, that for the desired treatment outcome the activity of export systems though the ABC transporters and capability to repair drugs’ caused DNA damage are essential. The worst prognosis observed for the carriers of all three unfavorable genotypes could be therefore an effect of, from the one hand the more efficient drugs and their metabolites efflux, and from the other hand improved DNA repair.

**Acknowledgements**

This work was financially supported by National Science Centre grant no. 2012/07/N/NZ5/00026.

## A6 Differences in gene expression in triple negative breast cancers associated with a better disease-specific survival of the hereditary cancer patients

### Edvins Miklasevics

#### Institute of Oncology, Riga Stradins University, Riga, Latvia

Hereditary triple-negative breast cancer patients have better disease-specific survival than sporadic ones. High expression of some of the miRNAs is related to worse overall and disease-free survival of triple-negative breast cancer patients. Thus, miR-214 showed significantly higher expression level in sporadic tissues than in hereditary ones (*p* = 0.0005). Triple-negative breast cancer patients with high level of miR-214 showed significantly worse disease-specific survival than patients with low level (*p* = 0.0314). Furthermore, comparison of global expression patterns between sporadic and hereditary breast cancer patients reveled a number differentially expressed genes. Expression of some of these genes - C12ORF23, C1ORF19, AMMECR1L - correlated with expression levels of miR-214. According to our results miR-214, and maybe some other miRs can be an indicator of the triple negative breast cancer patient’s poor prognosis and possibly could be used as a potential prognostic biomarker.

## A7 High incidence of BRCA1 mutations in patients diagnosed with breast cancer during pregnancy

### M. Szwiec^1^, J. Tomiczek-Szwiec^1^, M. Gełej^1^, C. Cybulski^2^, T. Huzarski^2^

#### ^1^Regional Oncology Center, Opole, Poland; ^2^Department of Genetics and Pathology, International Hereditary Cancer Center, Pomeranian Medical University, Szczecin, Poland

The aim of the study was to evaluate the frequency of recurrence mutations in the BRCA1 and BRCA2 genes in a group of patients consulted in Opole Oncology Centre diagnosed with breast cancer up to the age of 40 and in the group with breast cancer diagnosed during pregnancy in the years 2001-2014 as well as the assessment of clinical and histopathologic features of breast cancers identified in this group.

**Materials and methods:**

The analysis was performed in a group of 191 consecutive patients with breast cancer diagnosed up to the age of 40 consulted in Opole in Oncology Center between 2001 and 2014, who had a genetic consultation and tests to identify recurrent mutations in the BRCA 1 and BRCA2. We determined the incidence of breast cancer during pregnancy, clinical and histopathologic features of diagnosed cancers, week of pregnancy at the time of diagnosis, time and manner of termination of pregnancy, birth weight, presence of malformations of newborn and the incidence of recurrent mutations in BRCA1 and BRCA2 within the group of patients and in the group of patients diagnosed with breast cancer during pregnancy.

**Results:**

Within the group recurrent mutations in the BRCA1 (C61G, 4153delA, 5382insC, 185delAG 3875del4, 3819del5, 5370C> T) and BRCA2 (886delGT, 4075delGT, 5972C> T, 6174delT, 8138del5, 5467insT) was detected in 31 patients representing 16.2% of all (31/191). A mutation in the BRCA1 gene was detected in 29 patients (15.2%) and BRCA2 mutation in 2 patients (1.0%). The following mutations in the BRCA1 were found: 5382insC in 14 patients (7.3%), C61G in 6 patients (3.2%), 3819del5 in 6 patients (3.2%), 185delAG in 2 patients (1.0%), 5370C>T in 1 patient (0.5%). In the BRCA2 gene there was recognized 8138del5 and 5467insT each in one patient (0.5%). In the group of patients diagnosed with breast cancer in pregnant recurrent mutations in BRCA1 and BRCA2 genes were diagnosed in 5 from 14 patients (35.7%). There were detected only mutations in the BRCA1 gene, respectively C61G in 3 patients, 5382insC in 1 patient and 3819del5 also in one patient. Breast cancer related to pregnancy amounted 7,3% (14/191) of the whole group. The diagnosis during pregnancy was obtained in 8 patients (4.2%), and after delivery in 6 patients (3.1%). The average age of recognized breast cancer was 34.8 years old (28-39). Locally advanced diseases in stage III was observed in 7 patients (50.0%), overexpression of HER2 in 6 patients (42.8%), grade three tumor in 7 patients (50.0%), negative receptor status in 6 patients (42.8%) and in the majority of patients a high Ki-67 (range 20% -100%). Using immunohistochemical surrogates a positive HER2 subtype of breast cancer was observed in 6 patients, basal in 4 patients, luminal B in 3 patients and luminal A in 1 patient. In the case of patients with mutations in the BRCA1 gene we observed luminal B subtype in two patients, basal in two patients and luminal A in one. In 12 patients we evaluate the time from first symptoms to the start of the diagnosis the average was 11.4 weeks (range from 1 to 24 weeks). Delay in diagnosis was caused by the difficulties in clinical breast examination. Multidrug chemotherapy based on anthracycline during pregnancy received 5 patients who gave a birth between 35 and 41 week of gestation children of normal weight and no congenital anomalies. There was observed an increase in the number of patients with diagnosed breast cancer during pregnancy in seven-year intervals. Between 2001 to 2007, there were diagnosed four patients and in one of them mutations in the genes BRCA1 and BRCA2 were detected. Between 2008 to 2014 breast cancer in pregnancy was diagnosed in ten patients, and in four of them recurrent mutations in the BRCA1 gene were found.

## A8 Clinical observations during the course of breast cancer within family systems illustarted with an example of 5 diagnostic and therapeutic profiles

### E. Kilar

#### Department of Oncology, Disctict Specialist Hospital, Świdnica, Poland

As a clinical oncologist I have been treating patients with cancer for many years what enables me to observe repeatable regularities. Before the period of BRCA1 mutation assessment in Poland, during the treatment one can notice that:

1. there is a relation between the occurrence of some cancers within a family,
2. cancer occurs quite often at a very young age.

The development of genetics ent and DNA molecular research in determination of predisposition to malignant neoplasms give additional diagnostic tools crucial in planning medical intervention. My cooperartion with professor J. Lubiński's team from International Hereditary Cancer Centre in Szczecin and possibilities of BRCA1 mutation assessment since the year 2000 enable me to give some examples of diagnostics and treatment of families diagnosed with BRCA1 mutation. The quoted examples show that:

- in succeeding generations the disease may appear at a very early age (profiles 1 and 2),
- the cancers are both of a high grade and a high Ki-67 index, up to 100%,
- the receptor status on cancer cells is often negative,
- the course of the disease may be dynamic/aggressive (profile 4),
- long remissions/successful treatment may be achieved (profile 5),
- prophylactic treatment of adnexectomy (the removal of a lump in the tissue of the adnexa of the uterus) may give positive results (profiles 1, 2 and 5),
- diagnostic and therapeutic care for individuals with BRCA1 mutation diverges from recognised oncological standards,
- the analysed female patients are in the care of the Programme of the Ministry of Health – The medical care for individuals with inherited predispositons to cancers, and those who were oncologically diagnosed, are also in the care of oncological clinics (profile 4).

Five diagnostic and therapeutic profiles were analysed:

- Profile 1 - A mother and daughter diagnosed with breast cancer at the same time.
- Profile 2 - A mother, mother's sister and daughter diagnosed with breast cancer
- Profile 3 - A mother, mother's sister diagnosed with malignant breast cancer; prophylaxis for a daughter
- Profile 4 - A mother diagnosed with malignant breast cancer; prophylaxis for a daughter
- Profile 5 - Two women suffering from malignant neoplasm with BRCA1 mutation; long remissions/successful treatment.

It is quite interesting that young women both healthy, being conscious of the importance of BRCA1 mutation, and those who underwent oncological treatment, make decisions to start a family and have children.

## A9 Gene expression profile of MTC in relation to RET mutation status

### Małgorzata Oczko-Wojciechowska^1*^, Michał Świerniak^1,3*^, Jolanta Krajewska^1*^, Małgorzata Kowalska^1^, Tomasz Tyszkiewicz^1^, Agnieszka Pawlaczek^1^, Michał Jarząb^2^, Monika Kowal^1^, Dagmara Rusinek^1^, Jadwiga Zebracka-Gala^1^, Agnieszka Czarniecka^2^, Barbara Jarzab^1^

#### ^1^Department of Nuclear Medicine and Endocrine Oncology, Maria Skłodowska-Curie Memorial Cancer Center and Institute of Oncology, Gliwice Branch, Gliwice, Poland; ^2^III Department of Radiotherapy and Chemotherapy, Maria Skłodowska-Curie Memorial Cancer Center and Institute of Oncology, Gliwice Branch, Gliwice, Poland; ^3^Genomic Medicine, Medical University of Warsaw, Warsaw, Poland

^*^Authors contributed equally

**Aim of the study**

Medullary thyroid cancer (MTC) arises from parafollicular C and occurs as hereditary (20% of all cases) and sporadic form. Hereditary type is a consequence of RET protooncogene germline mutations. Strong genotype-phenotype manifestation is correlated with different sites of RET mutation. The aim of study was to evaluate whether the differences in gene expression profile are related to the particular RET mutation.

**Methods**

Fresh-frozen tumor samples from 60 MEN 2 patients and 21 RET negative MTC patients were collected. Germline mutation screening was performed according to standard diagnostic algorithm. RET somatic mutations were analyzed among sporadic MTC patients and twenty one exons of RET gene were directly sequenced. Gene expression profile was analyzed in 34 MTC samples using Gene Chip 1.0 ST Arrays (Affymetrix). An independent set of 26 MTC samples was used for QPCR validation.

**Results**

Hierarchical clustering did not show any global differences in gene expression profile between type of RET mutation, however, supervised analysis revealed 10 genes differentially expressed between tumor samples with mutation at RET codon 634 and 918. We select 5 genes for QPCR validation and confirmed 3 of them as deregulated. Additionally we also did not find significant changes between hereditary and sporadic MTC.

**Conclusion**

The differences in gene expression profile of slightly are dependent of RET site mutation.

**Acknowledgements**

Supported by NCN, grant no NN401 4106 39

## A10 Detection method for the 3’EPCAM genomic deletion and its frequency in Polish HNPCC patients

### Andrzej Plawski^1^, Paweł Borun^1^, Joanna Szczepinska^1^, Monika Siolek^2^, Beata Kozak-Klonowska^2^

#### ^1^Institute of Human Genetics, Polish Academy of Sciences, Poznan, Poland; ^2^Department of Genetics, Holycross Cancer Centre, Kielce, Poland

Lynch syndrome is a frequent, autosomal, dominantly-inherited cancer predisposition syndrome caused by various germline alterations that affect DNA mismatch repair genes, mainly MLH1, MSH2 and MSH6. EPCAM (Epithelial Cell Adhesion Molecule) large rearrangements, localized upstream of MSH2 on chromosome 2, have been recently described as the one of the genetic factors associated with the Lynch syndrome occurrence. 3’EPCAM genomic rearrangements may be a cause of mismatch repair deficiency in some Lynch syndrome families. The aim of the study was to develop cost-effective screening tool for the rapid detection of 3'EPCAM genomic rearrangements, along with its validation, and determination of the 3'EPCAM genomic rearrangements status in our group of HNPCC patients. We applied developed by us C-HRM method enable to detect CNVs of the 3'EPCAM and simultaneously screen for small mutation in two exons of the MLH1 gene containing small mutation hot-spots. In our group of 250 Lynch syndrome probants we detected 2 cases of 3'EPCAM genomic rearrangement and 4 small mutations within the MLH1 gene.

## A11 Selected micro- and macroelements as risk factors of cancers, prospective observation studies: Zinc

### Katarzyna Kaczmarek^1^, Magdalena Muszyńska^2^, Wojciech Marciniak^2^, Grzegorz Sukiennicki^1^, Marcin Lener^1^, Katarzyna Durda^1^, Katarzyna Jaworska-Bieniek^1^, Tomasz Gromowski^1^, Tomasz Huzarski^1^, Tomasz Byrski^1^, Jacek Gronwald^1^, Oleg Oszurek^1^, Cezary Cybulski^1^, Tadeusz Dębniak^1^, Antoni Morawski^2^, Anna Jakubowska^1^, Jan Lubiński^1,2^

#### ^1^Department of Genetics and Pathology, International Hereditary Cancer Center, Pomeranian Medical University, Szczecin, Poland; ^2^Read – Gene, S.A., Grzepnica, Poland

##### **Correspondence:** Katarzyna Kaczmarek (katarzyna.kaczm@gmail.com)

**Background**

Zinc is a micronutrient, which is essential for human health, playing role as a cofactor of enzymes such as dehydrogenases, peptidases and component of zinc finger domains. Recently, it has been reported that zinc may play role in chemoprevention, and its level may be associated with occurrence of cancers. Depending on the study, patients affected with cancer have increased or decreased serum Zn levels comparing to controls.

**Aim of the study**

The aim of the study was to evaluate the relationship between zinc blood levels and subsequent cancer risk in a large cohort of persons followed for incident cases of cancer in Szczecin Poland, using a case-control study design.

**Material and methods**

Study group was selected from among persons whose biological material is biobanked in our center. In biobank we collected samples from ~26 000 people with no previous cancer diagnosis at the time of blood collection. All patients were followed for cancers diagnosis. Study group was divided into 3 subgroups: 69 men with all sites cancers and 138 unaffected controls, 102 women with all sites cancers (BRCA1 mutation carriers exclude) and 204 unaffected controls, 87 women with BRCA1 mutation and breast/ovarian cancer and 174 controls.

Zinc level in serum was measured by inductively coupled plasma mass spectrometry (ICP-MS) using Elan DRC-e ICP-Mass Spectrometer, Perkin Elmer. For statistical analysis individuals in each group were divided into 4 quartiles. We performed comparison of number of cases and controls in each quartile. OR was calculated using Fisher’s exact test.

**Results**

It was observed that men with Zn level >997 μg/l have a tendency to decreased risk of cancer comparing to men with level <997 μg/l. (OR=0,61, *p*=0,23, CI=0,2984-1,241).

Women without BRCA1 mutation with Zn level >921μg/l have a tendency to decreased risk of cancer comparing to women with level <921μg/l (OR=0,62, *p*=0,06, CI=0,3787-1,101).

BRCA1 mutation carries who underwent previous adnexectomy and with Zn level >976 μg/l have a tendency to decreased risk of cancer comparing to women with level <976 μg/l. (OR=0,59, *p*=0,46, CI=0,210-1,672).

BRCA1 mutation carries with no adnexectomy and with Zn level 897,95–995,71 μg/l have a tendency to decreased risk of cancer comparing to women with 499,08–786,41 μg/l. (OR=0,57, *p*=0,34, CI=0,2224-1,456).

**Conclusions**

Results from this study suggests that serum zinc concentration may be associated with cancer risk.

**Acknowledgments**

This project was financially supported by National Science Centre, grant no. 2012/07/N/NZ4/02433.

## A12 Selected micro- and macroelements as risk factors of cancers, prospective observation studies: Iron

### Grzegorz Sukiennicki^1^, Magdalena Muszyńska^2^, Wojciech Marciniak^2^, Katarzyna Kaczmarek^1^, Marcin Lener^1^, Katarzyna Durda^1^, Katarzyna Jaworska-Bieniek^1^, Tomasz Gromowski^1^, Tomasz Huzarski^1^, Tomasz Byrski^1^, Jacek Gronwald^1^, Oleg Oszurek^1^, Cezary Cybulski^1^, Tadeusz Dębniak^1^, Antoni Morawski^2^, Anna Jakubowska^1^, Jan Lubiński^1,2^

#### ^1^Department of Genetics and Pathology, International Hereditary Cancer Center, Pomeranian Medical University, Szczecin, Poland; ^2^Read – Gene, S.A., Grzepnica, Poland

##### **Correspondence:** Grzegorz Sukiennicki (gsukiennicki@wp.pl)

**Background**

Iron plays an important role in many metabolic processes, is included in the delivery of oxygen to cells and redox processes. Deficiency and excess of iron can lead to multiple organ failures and in extreme cases to death. The latest studies show that the iron can significantly influence the risk of cancer development and progression. Scientists reported association between high levels of iron in the serum and the risk of colon, liver, stomach and breast cancers. The low iron level was detected in patients with bladder and lung cancers.

**Aim**

The aim of the study was to find a possible correlation between iron blood levels and cancer risk in a large cohort of persons followed for incident cases of cancer in Szczecin Poland, using a case-control study design.

**Material and methods**

Study group was selected from among persons whose biological material is biobanked in our center. Serum samples were stored at -80^o^ C until the iron assay was conducted. In biobank we collected samples from ~26 000 people with no previous cancer diagnosis at the time of blood collection. All patients were followed for cancers diagnosis. Study group was divided into 3 subgroups: 69 men with all sites cancers and 138 unaffected controls, 102 women with all sites cancers (BRCA1 mutation carriers exclude) and 204 unaffected controls, 87 women with BRCA1 mutation and breast/ovarian cancer and 174 controls.

Zinc level in serum was measured by inductively coupled plasma mass spectrometry (ICP-MS) using Elan DRC-e ICP-Mass Spectrometer, Perkin Elmer. For statistical analysis individuals in each group were divided into 4 quartiles. We performed comparison of number of cases and controls in each quartile. OR was calculated using Fisher’s exact test.

**Results**

Men with Fe serum level 1450-1900 μg/l had 5 times lower risk of cancer than other men (OR =0,24 *p* =0,021). Women without BRCA1 mutation and with Fe level 900-1200 μg/l had more than 2 times lower risk of cancer comparing to the rest (OR =0,48 *p* =0,012). BRCA1 mutation carries with adnexectomy and with Fe level 1050-1350 μg/l had nearly 8 times lower cancer risk than women wit level <1050 μg/l (OR =0,13 *p* =0,0037). BRCA1 mutation carries without adnexectomy and with Fe level 1400-1900 μg/l had more than 3 times lower risk of cancer than other women (OR=0,29 *p* =0,051).

**Conclusion**

Our study suggests that serum iron level may be risk factor for cancer.

## A13 Is serum selenium concentration associated with the development of age-related cataract?

### Michał Post

#### Department of Ophthalmology, Pomeranian Medical University, Szczecin, Poland

**Purpose**

To evaluate the correlation between the occurrence of age-related cataract and the concentration of serum selenium.

**Methods**

The study group included 95 cataract patients, aged 56-89 years, not suffering from other diseases of the eye nor systemic diseases with proven impact on the eye function/electrolytes. Control group consisted of 187 healthy subjects. Selection criteria for the control group were: sex, age, smoking. People taking supplements were excluded from the study. Measurement of the serum selenium concentration was carried out by Inductively Coupled Plasma Mass Spectrometry (ICP-MS). The statistical analysis was performed by Fischer’s exact test.

**Results**

1. The lower levels of selenium were associated with greater occurrence of cataract,
2. The threshold point of selenium was ~70 μg/l (female) and ~73 μg/l (male) for an increase in age-related cataract occurrence

**Conclusions**

1) The concentration of selenium in the blood may be a marker of occurrence of age-related cataracts. 2) The low selenium levels may be a risk factor for age-related cataract in the Polish population

**Keywords**

selenium, age-related cataract

